# Supplementary material for: LINC01270 Regulates the NF-κB-Mediated Pro-Inflammatory Response via the miR-326/LDOC1 Axis in THP-1 Cells
Source: Cells. 2024 Dec 8;13(23):2027. doi: 10.3390/cells13232027 (PMC11640305; doi:10.3390/cells13232027)
Supplement: Supplementary file 1 [file cells-13-02027-s001.zip › cells-3344999-supplementary.pdf]

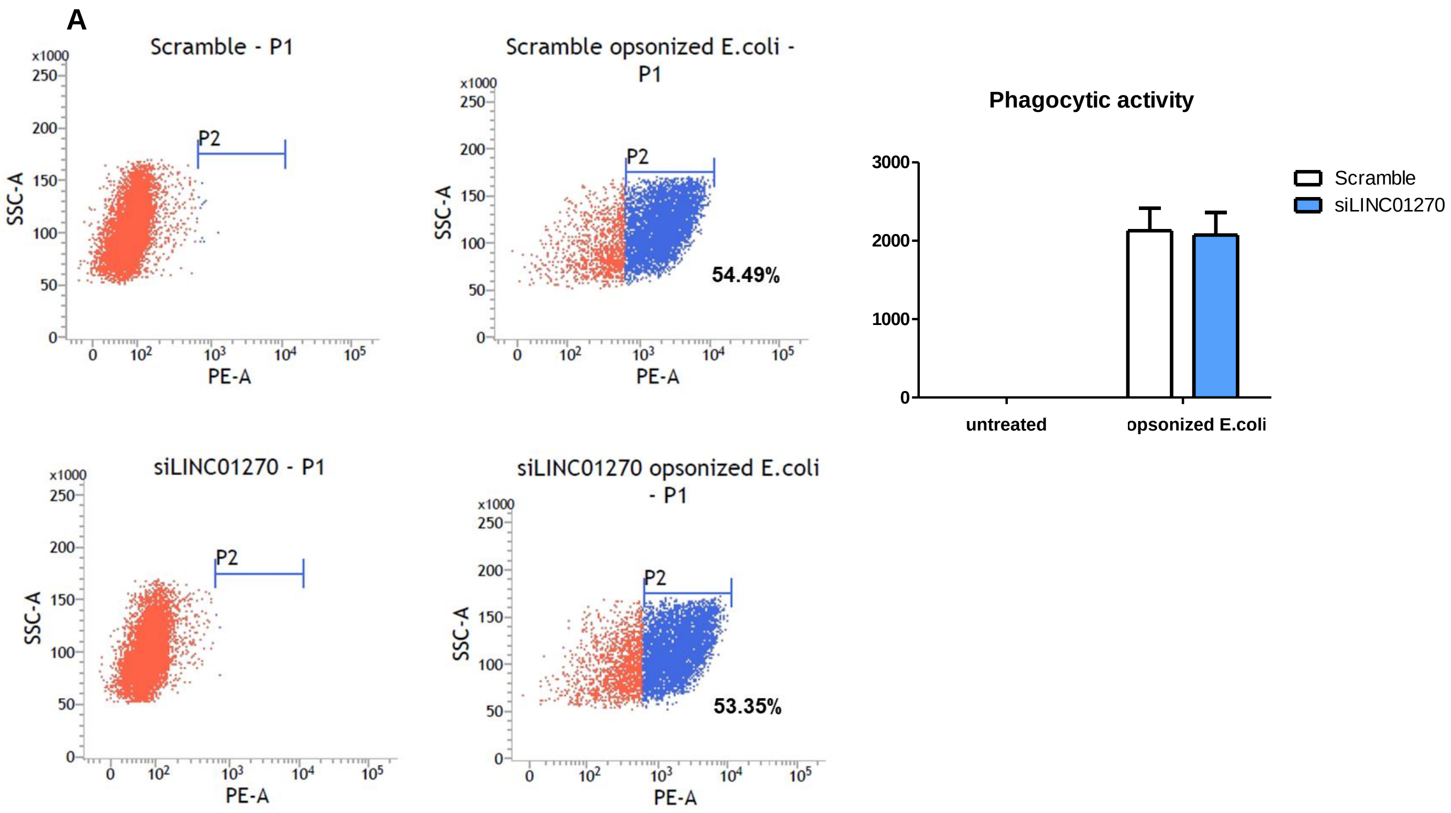

Figure S1. LINC01270 attenuation has no effect on phagocytic activity of THP-1 cells. (A) Cells transfected with either scramble or siLINC01270 were treated with opsonized *Escherichia coli* (strain K-12) BioParticles at a concentration of 20μg/ml for 3h. Phagocytic activity was measured by flow cytometry.

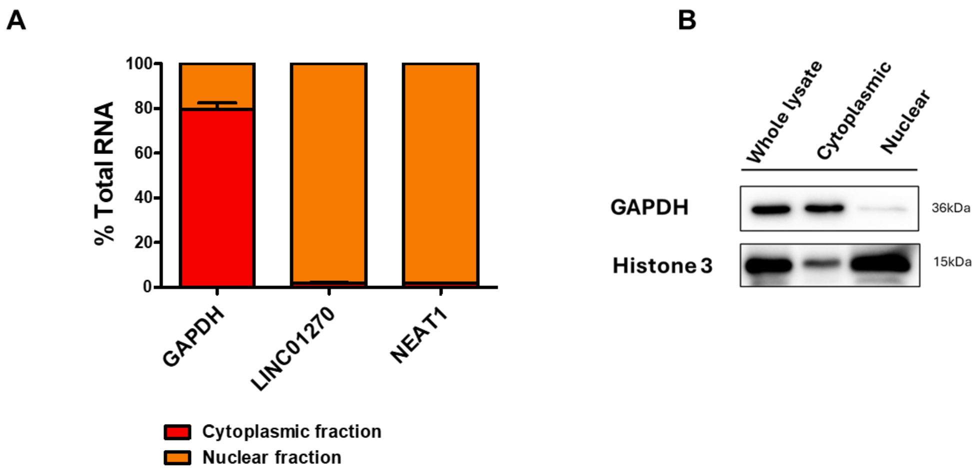

Figure S2. LINC01270 is localized in the nucleus in THP-1 cells. (A) Nuclear and cytoplasmic fractions of THP-1 were analyzed via qPCR (Nuclear positive marker :NEAT1, cytoplasmic positive marker: GAPDH). (B) Western blot of nuclear and cytoplasmic fraction of THP-1 cells qPCR (Nuclear positive marker :Histone 3, cytoplasmic positive marker: GAPDH).

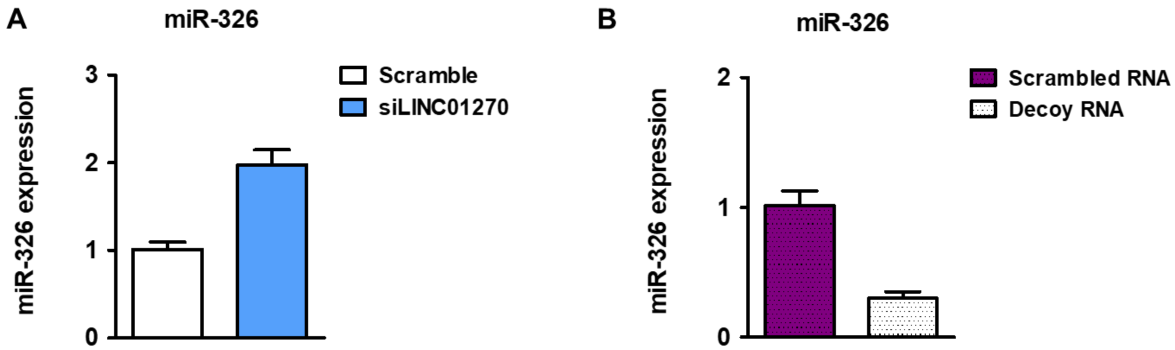

Figure S3. LINC01270 sponges miR-326. (A) miR-326 expression measured via qTR-PCR in scramble or siLINC01270 transfected THP-1. (B) miR-326 expression measured via qTR-PCR in THP-1 cells transfected with scrambled RNA or a decoy RNA containing the miR-326-binding site of LINC01270.

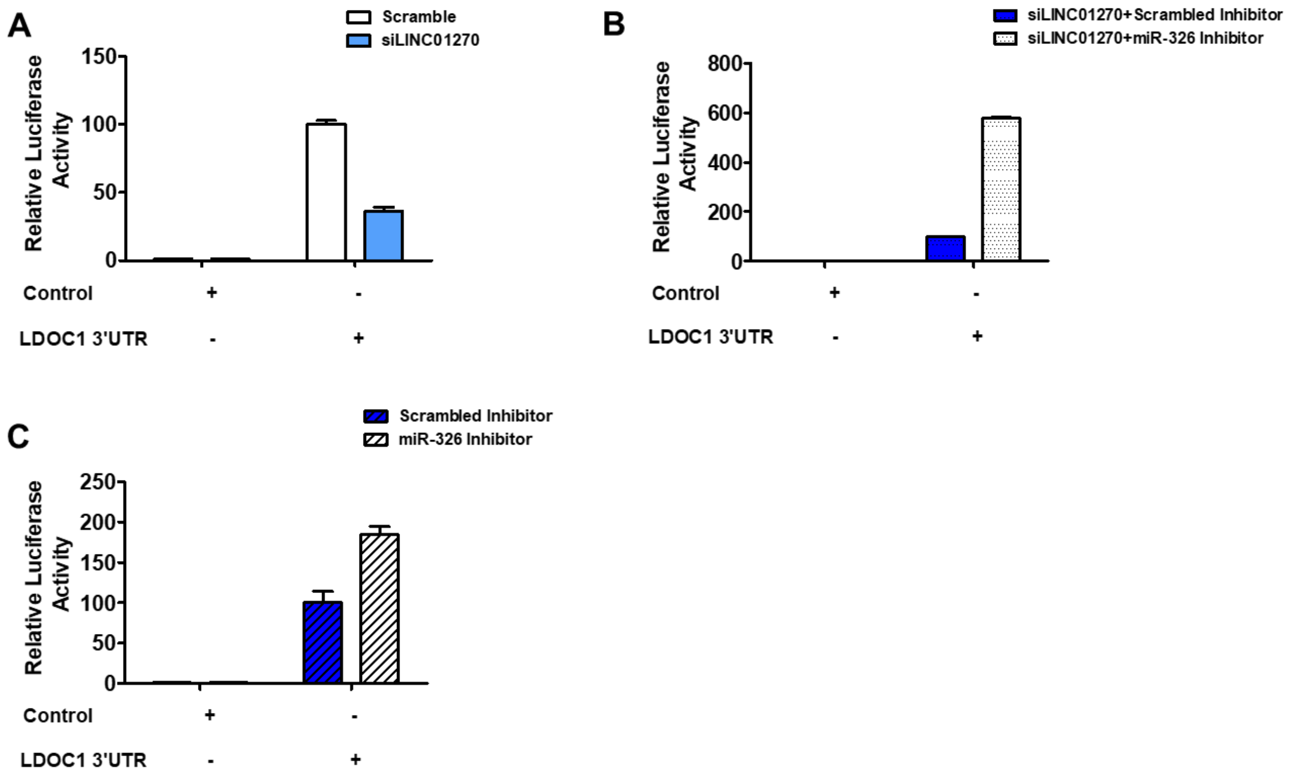

Figure S4. miR-326 sequester LDOC1 mRNA. (A) HEK293 cells were transfected with scramble or siLINC01270 (B) or co-transfected with siLINC01270 and either scrambled inhibitor or miR-326 inhibitor, the LDOC1 3'UTR luciferase activity was measured by a dual luciferase assay. (C) Scrambled or miR-326 inhibitors were transfected in HEK293 cells, LDOC1 3'UTR reporter luciferase activity was measured by a dual luciferase assay.
